# Influence of Rehabilitation Aid with Biofeedback on the Rehabilitation Process during Remote Home-Based Rehabilitation

**DOI:** 10.3390/ijerph19159069

**Published:** 2022-07-25

**Authors:** Mariana Zadrapova, Eva Mrázková, Miroslav Janura, Michal Strycek, Martin Cerny

**Affiliations:** 1 Department of Epidemiology and Public Health, Faculty of Medicine, University of Ostrava, 70300 Ostrava, Czech Republic; mariana.zadrapova@fno.cz (M.Z.); eva.mrazkova@osu.cz (E.M.); 2 Clinic of Rehabilitation and Physical Medicine, University Hospital of Ostrava, 70800 Ostrava, Czech Republic; 3 Department of Natural Sciences in Kinanthropology, Faculty of Physical Culture, Palacky University Olomouc, 77147 Olomouc, Czech Republic; miroslav.janura@upol.cz; 4 Department of Rehabilitation, Faculty of Medicine, University of Ostrava, 70300 Ostrava, Czech Republic; 5 Department of Cybernetics and Biomedical Engineering, Faculty of Electrical Engineering and Computer Science, VSB—Technical University of Ostrava, 70800 Ostrava, Czech Republic; michal.strycek@vsb.cz

**Keywords:** home rehabilitation, balance training, biofeedback, therapeutic telemedicine, knee joint

## Abstract

Ensuring the regularity and correctness of rehabilitation exercises in the home environment is a prerequisite for successful treatment. This clinical study compares balance therapy in the home environment on a conventional balance mat and an instrumented wobble board, with biofeedback supported by a rehabilitation scheme realized as web-based software that controls the course of rehabilitation remotely. The study included 55 patients with knee injuries. The control group consisted of 25 patients (12 females and 13 males, mean age 39 ± 12 years) and the study group of 30 patients (19 females and 11 males, mean age 40 ± 12 years). Treatment effects were compared using the ICS Balance Platform measurement system. Measurements showed significant differences in the change in ICS Balance platform parameters representing the dynamic stability of the patients. The dynamic stability improved more with the instrumented wobble board. The study did not show an influence of different methods of communication with patients during home-based rehabilitation.

## 1. Introduction

Knee injuries are one of the most common sports injuries [1]. Damage to the soft tissues in the joint results in altered proprioception, which affects the level of physical activity, balance, and muscle strength and has an impact on the risk of re-injury [2]. Inadequate neuromuscular control commonly occurs up to 2 years after anterior cruciate ligament (ACL) replacement [3].

Therapies on different types of balance devices are included in the rehabilitation process after knee injury [4] to enable the development of new sensorimotor strategies, facilitate functional mobility, and restore effective coordination. These therapies accelerate the onset of muscle contraction and mutual muscle coactivation and coordination, allowing the patient to respond better to unusual and sudden stimuli from the external environment and reducing the risk of injuries and falls. 

The rehabilitation protocol includes both working with the patient in a medical facility and ongoing self-therapy in the home environment without the supervision of a physiotherapist [5]. During physiotherapy, the therapist educates the patient about self-therapy, indicates appropriate exercises, points out possible errors, and continuously checks the correctness of their performance. Home therapy can speed up recovery by allowing patients to exercise at home between or after they have finished rehabilitation sessions. However, it must be appropriately monitored, as incorrect or imperfect therapy can adversely affect the patient’s recovery [6]. 

Modern information and communication technology, working on the principle of biofeedback, can be used for better control of self-therapy in the home environment. In addition to the summation of sensory stimuli, a benefit of biofeedback is increased attractiveness of the exercise and greater patient motivation. Biofeedback has been used in rehabilitation for more than 50 years to facilitate proper movement patterns after injury [7,8]. Biofeedback systems can provide important information about technique and exercise quality, allowing for real-time correction of movement [8].

Home rehabilitation equipment should meet the requirements of minimal wearable components, ease of use, minimal psychological burden, and affordability [9]. Correia et al. [10] compared home rehabilitation under regular supervision of a physiotherapist and home-based biofeedback therapy following knee replacement and concluded that therapy using digital biofeedback achieved better results than conventional home therapy. In contrast, Rhim et al. [11] demonstrated that supervised rehabilitation can provide benefits in improving muscle strength, neuromuscular control, and knee function compared to home rehabilitation up to 1 year after ACL repair. Although patients undergoing home rehabilitation may achieve acceptable levels of knee function and stability within 1 year, quality education may be required for patients who choose not to undergo supervised rehabilitation [11].

Despite best efforts, rehabilitation in the home may lead to a reduced rehabilitation effect due to irregularity of therapy, poor execution of exercises, or insufficient number of repetitions. Non-adherence to and poor performance of home rehabilitation leads to the need for exercise frequency monitoring and correct execution of movement therapy in the home environment [8,12]. Lack of supervision may lead to a reduction in the resulting effect [13]. We can ensure quality home therapy by monitoring movement and its correction by the user or by monitoring via an online or offline therapist [14,15,16]. Some studies have evaluated real-time monitoring exercise by either the therapist or the user themselves. 

One way to simultaneously provide feedback and a labile environment during therapy is to use a balance wobble board with built-in accelerometers, with the ability to connect to a computer or other Smart device to display the balance status in real time [17,18]. Wobble boards with the ability to display the movements performed on screen are affordable, portable, and user-friendly devices with the ability to objectify the dynamic balance performance of individuals outside the laboratory environment [17].

The commercially available biofeedback wobble board does not allow setting up your own set of exercises and also immediate control of the exercises performed by the physiotherapist remotely. Simultaneously, they often require the installation of special software in conjunction with a personal computer. Therefore, for the purpose of this study, we developed a measurement system for tracking the movements of balance rehabilitation aids and the possibility of remotely controlling the performed exercises. We chose a custom set of rehabilitation exercises with gradually increasing difficulty based on the success of previous rehabilitation exercises. We tested this system on a group of patients to determine the effect of using our system in a home-based rehabilitation setting.

## 2. Material and Methods

A total of 61 patients were enrolled after knee distortion, knee arthroscopy, or ACL plastics; 55 completed the study during the 3-week period in which they received therapy. Overall, 1 patient did not complete the study because of surgery, and 5 patients left the study without any explanation. The control group of patients was recruited first.

The control group consisted of 25 patients (12 female and 13 male, mean age 39 ± 12 years). In total, 16 patients had surgery (6 arthroscopies, 10 ACL). The mean time since surgery was 22 weeks (range 10–30 weeks). Overall, 9 patients did not have surgery and exhibited post-knee distortion. Mean time since injury was 27 weeks (range 8–66 weeks). The study group consisted of 30 patients (19 females and 11 males, mean age 40 ± 12 years). In total, 19 patients had undergone surgery (9 arthroscopies, 10 ACL). The mean time since surgery was 26 weeks (8–47 weeks). In total, 11 patients had not undergone surgery and exhibited post-knee joint distortion. Mean time since injury was 30 weeks (8–53 weeks).

### 2.1. Experimental Wobble Board with Biofeedback

**Figure 1:**
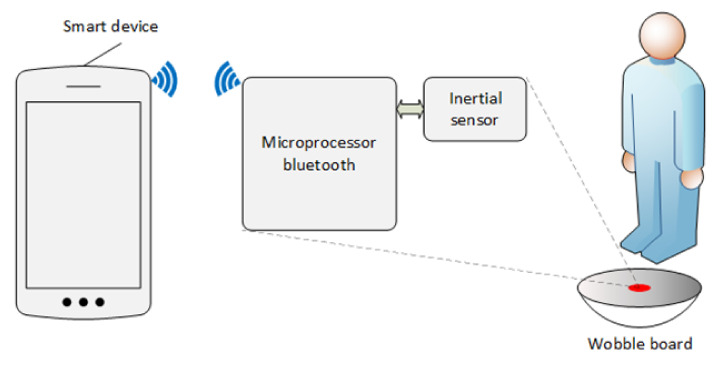
Experimental wobble board setup.

For the purpose of testing, a custom biofeedback rehabilitation tool was developed and described in detail elsewhere [19]. It consists of a wooden wobble board equipped with an inertial sensor and wireless communication with a device for displaying the progress of the rehabilitation exercise (Figure 1). The inertial sensor implemented in the circular section allows measurement of the inclination and rotation of the wobble board. The progress of the exercise is visualized for the patient in a web application that can be run on any device with a web browser, such as a smart device in the form of a television, tablet, or personal computer. By storing the actual measurement data on the web server, it allows the treating physiotherapist to monitor and analyze the success of the exercises performed while changing the difficulty or sequence of exercises performed for the next session.

### 2.2. Airex

The control group was provided with an Airex foam pad 48 cm × 40 cm × 6 cm in size (AIREX Swiss, Sins, Switzerland). The surface of the pad is both non-slip and stimulating. The foam technology has destabilizing properties that stimulate the activation of muscle stabilizers in the joint area. The thickness of the aid determines the degree of lability. Soft material alters proprioceptive input from the lower limb. Standing on a pliable surface induces body instability in the sagittal and frontal planes and alters efferent information to joint receptors and skin mechanoreceptors in the foot [20].

This aid was chosen because of its common use in the treatment of instability, and it is recommended in our departmental guidelines.

### 2.3. ICS Balance Platform

To measure balance and postural control, center of pressure (CoP) measurement using pressure platforms is considered the gold standard [16,17,21,22]. To validate the measurements, the ICS Balance Platform system (Otometrics, Taastrup, Denmark) was used, which is a stability platform that enables the measurement of static and dynamic stability parameters. For the purpose of this study, the module ‘Balance training’ was used. This module is a set of diagnostic exercises defined by the manufacturer. It served as a good indicator of the progress in stability and includes two dynamic exercises in addition to other static exercises commonly used in the ICS Balance Platform system. 

### 2.4. Data Collection

Measurements began in December 2019 and concluded in May 2021. Each patient was tested on the ICS Balance Platform before starting rehabilitation in the home setting. Baseline anthropometric data and anamnestic data were obtained simultaneously (i.e., patient’s age, height, and weight). In addition, we obtained data on injuries and surgeries, sports, work and leisure history, and information on the presence of migraines, headaches and dizziness, and balance disorders. The knee joint status questions were: presence or absence of rest pain, occasional pain, pain after physical strain, knee instability, limitation of knee joint range of motion, and stiffness. This included information on whether or not rehabilitation had taken place and, if so, whether it had been completed or was still ongoing.

This was followed by home rehabilitation therapy, which lasted for 3 weeks. The control group received an Airex balance mat and instructions with 16 recommended balance exercises of increasing difficulty. These were exercises that are commonly performed on balance pads, including weight bearing exercises, mat loading, walking on the mat, and squats (https://my-airex.com/, accessed on 5 July 2019). The choice of exercises with Airex in the home environment was made by the individual. Patients were instructed not to provoke pain and a feeling of instability during home exercises. The recommended exercise frequency was 5 times a week for 15 min. 

**Figure 2:**
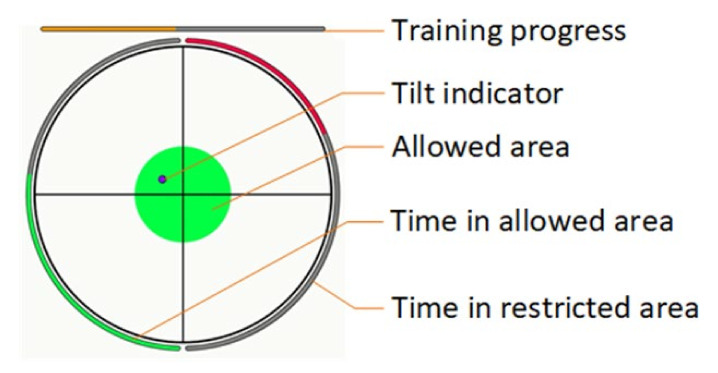
User interface for the experimental wobble board.

The study group performed the therapy on the experimental wobble board. The recommended frequency of exercise was 5 times per week, with a total therapy time of approximately 15 min. The rehabilitation training was guided by software that showed the patient’s progress during the exercise (Figure 2).

### 2.5. Experimental Protocol

Each patient was educated in the same manner before the home therapy. Afterwards, the study group was divided into three groups depending on the communication strategy. The first group (Educated group, 8 patients) was only educated, as described above. Each of them received a phone number with instructions that, in case of any problem or discomfort, they could contact the rehabilitation specialist. Nobody used this number to contact them. The second group (Controlled group, 11 patients) was educated and contacted by phone by the rehabilitation specialist once during the first week of home rehabilitation. They were asked if the device works well, if they are happy with the rehabilitation schedule, and if they have any problems to solve. There were no identified complaints or requests for further education. The last group (Assisted group, 11 patients) were educated and contacted by phone the same way as the second group. The technical solution of the wobble board allows the number of started exercises to be controlled remotely by the web interface. Patients were contacted by phone by the rehabilitation specialist if they had not started at least 70% of the planned exercises in the past 5 days. They were asked if they were facing any technical problem or had any other limitations that did not allow them to continue with rehabilitation. We contacted 3 patients in total. Overall, 2 of them were less motivated and 1 had a problem with the internet connection. 

Rehabilitation training consists of five types of exercises and a break. These are sequenced in increasing difficulty with each successful completion of all exercises of the same type within one rehabilitation session. Each session is followed by a 30 s break. The user can come down from the section and rest. The zone is defined around the circle. The total number of breaks is 12.

Each rehabilitation session consisted of the following sequence of exercises:

1. Stability—the user must stay within the allowed area for 10 s. The area is in the shape of a circle that is placed in the middle of the tilt limits and, as the difficulty increases, the circle decreases from the initial 4 cm to the final 1.5 cm. The number of sessions is 6. The specified time for failure to complete the task is 20 s.
2. Segments—the user must stay within the allowed area for 5 s. The area is in the shape of a quarter circle. This exercise has no defined difficulty. The number of sessions is 8. The defined time for failure to complete the task is 15 s.
3. Cross stability—the user must stay within the allowed area for 3 s. The area takes the form of a rectangle intersected by a circle (one of the longer sides is convex) on one of the axes of the tilt limits and becomes proportionally smaller as the difficulty increases. The total number of sessions is 16. After four sessions, the zone is reduced.
4. Cross reach—the user must touch/reach the allowed area. The area takes the shape of a small square on one of the axes of the tilt limits and moves away from the center as the difficulty increases. The total number of touches is 24.
5. Moving in a circle—the user must touch/reach the allowed area. The area is in the shape of a small circle, which is placed on a concentric circle between the inclines. The session is divided into four sets of exercises between which breaks are inserted. In the first session, the areas follow a clockwise direction. In the second session, the areas appear randomly. The third and fourth sessions are completed with an area in the center of the surface. After reaching the area on the circle, it is necessary to return to the base position. The difference between the third and fourth sessions is the random appearance of the areas. The specified time for failure to complete the task (touch) is 10 s.

After completion of rehabilitation in the home environment, each patient was tested on the ICS Balance Platform.

### 2.6. Data Structure

The following parameters of the Balance training module were further evaluated from the control measurements on the ICS Balance Platform, which contains four static and two dynamic measurements. The static measurements occurred 30 s and resulted in the average rate of change in the center of gravity position detected by the stability platform (mm/sec): EO, standing on the platform with eyes open; EC, standing on the platform with eyes closed; OLT, standing on one lower limb; FC, standing on an unstable surface with eyes closed. Dynamic measurements occurrated 120 s: Tar and Purs. For Tar, the patient is asked to tilt his/her body on the stability mat to follow a target that gradually appears at different locations on the screen. When the target is reached, a new target appears, which gradually becomes smaller and further away. The measured parameter is the number of successful touches of the target in relation to the duration of the test (hits). For Purs, the patient is asked to keep the indicator of their body’s center of gravity, as measured by the stabilometric platform, within the area of a circular point that moves in a spiral, changing its size and gradually increasing its speed of movement. The parameter measured is the average distance of the body’s center of gravity relative to the center of the moving circular point for the duration of the test in millimeters.

All measurements performed within the Balance training module on the ICS Balance Platform are represented in measured values according to the above definitions and at the same time on a point scale (ICS Balance Score), which recalculates the measured values according to the algorithms defined by the manufacturer of the platform. All monitored parameters are also evaluated on this scale and are marked analogously with the abbreviations above with the addition of the points—P. The Balance training module sums the recalculated values and interprets them as the total score of the Balance training module (Tot_P).

### 2.7. Statistical Analysis

Normality was tested using the Shapiro–Wilk test. Based on the results, the corresponding pair tests were selected. Student’s *t*-test was used for differences in the tested parameters that had normal distributions and passed the test of agreement of variances, whereas Welch’s *t*-test was chosen for the tested parameters that did not pass the test of agreement of variances. Parameters that did not have a normal distribution were tested using the Mann–Whitney U test. Comparisons were made at the significance level of α = 0.05. For analysis of variance, we used the ANOVA.

## 3. Results

### 3.1. Questionnaire

The anthropometric data and anamnestic data showed that the feeling of instability was predominant in 18 patients in the study group and 15 patients in the control group. Occasional knee pain was experienced by 27 patients in the study group and 22 patients in the control group. The feeling of stiffness or limitation of range of motion, as well as rest pain or pain after loading, were also similar between the two groups (stiffness, 14 vs. 11; limitation of range of motion, 12 vs. 10; rest pain, 5 vs. 6; and pain after exertion, 22 vs. 20 in the study vs. control, respectively). The majority of patients had received a physiotherapy intervention in the past but not in treatment of monitored diseases. Only 10 participants had not attended supervised physiotherapy. Based on this questionnaire research, there was no difference between the control and study groups in terms of subjective assessment of the knee joint.

### 3.2. Measurements

In the first step of evaluating the measured data using the ICS Balance Training system, we verified no significant difference between the parameters measured before the start of home rehabilitation between the two groups. The study group was not divided into separate groups based on the communication strategy because the mean number of successfully finished exercises per patient was very similar (13.8 ± 0.6 finished exercises).

Subsequently, a calculation was performed for each ICS parameter to determine the difference between before and after the home-based rehabilitation phase. Negative mean values of the parameter with the suffix _P means that after the end of the rehabilitation in the home environment, the patient achieved a worse result in the given test—they achieved a lower number of points. This happened with the parameters EO_P, EC_P, OLT_P, which also have significant values of standard deviation. The EC_P parameter acquires significantly more negative values in the control group than in the study group. Therefore, we can define that the study group achieved better results in this test. Analogously to these conclusions, we interpret values for parameters without the suffix _P (Table 1).

Significant differences were found after home rehabilitation between the study and control groups for the parameters Tar (*p* < 0.001), Purs (*p* = 0.010), and EC (*p* = 0.020). When these parameters were converted into a scoring scale by the ICS Balance Platform system, significance was maintained for Tar_P (*p* < 0.001) and Purs_P (*p* = 0.010). P_Tot, which represents the overall result of the ICS Balance Platform assessment, was significantly different (*p* = 0.019) between the study and control groups. The difference in the mean values of this parameter was 34.3 points.

Based on the overall results, we further analyzed parameters EC, Tar, and Purs in the study group depending on the communication strategy (Table 2). The analysis based on ANOVA tests showed no significant difference in these parameters between these different groups.

## 4. Discussion

Previous research has shown that balance training is an effective means of improving static and dynamic stability in resting, proactive, and reactive balance [23]. The aim of our study was to compare the effect of balance therapy on a commonly used soft mat and an experimental wobble board supplemented with biofeedback in the home environment. We confirmed the positive effect of both rehabilitation aids. Tot_P, representing the total score of the ICS Balance Platform, was experimentally set as an indicator of stability. It is commonly used in stability diagnosis. Both groups (study and control) had an increase in this parameter after rehabilitation. However, when comparing Tot_P before and after home rehabilitation for each group, the mean value of the study group was 34.3 points higher than that of the control group. A reduction in the variance of this parameter in the study group was also evident (STD_study group = 46.91 points, STD_control group = 58.67 points). Based on these results, we concluded that using the wobble board is more effective during home-based rehabilitation. 

When analyzing the individual parameters of the ICS Balance Platform from which Tot_P is calculated, we found that most of these parameters were not significantly different (see Table 1). The only significant difference between the control and study groups was observed in Tar and Purs (*p* < 0.001 and *p* = 0.010, respectively) in favor of the study group. Both of these parameters represent dynamic stability improvement.

We should take into account the influence of biofeedback, in this case the influence of eye control during testing with the ICS Balance Platform. The study group was trained with visual biofeedback. Changing proprioception has many consequences; it affects physical activity level, balance ability, and muscle strength and increases the risk of repetitive strain injury [2,24]. There are two similar static tests of stability, one with open eyes (parameter EO) and one with closed eyes (parameter EC). The statistical analysis showed significant differences in EC. The control group had slight improvement in the EC test, but there were no significant changes in the study group. Standing on an unstable platform leads to changes in sensory biofeedback and patients become more dependent on visual information [25], with the spherical stretch being more unstable than the soft balancing platform; therefore, we expect a greater dependence on visual support.

Our study also focused on different approaches in communicating with patients. At the beginning, all patients were educated in the same way, but, during the home-based rehabilitation, their communication with the rehabilitation professional was slightly different (Educated, Controlled, and Assisted groups). We did not observe differences in the number of finished exercises between these groups, and we did not find any significant differences in the monitored ICS Balance Platform parameters between these three groups (see Table 2). Other studies have reported a positive impact of physiotherapists taking a more active approach during remote-based rehabilitation [26], but we could not confirm this in our study. The reason may be the limited number of participants or because of lifestyle changes caused by the COVID-19 pandemic.

The benefit of the experimental wobble board, in addition to providing an unstable environment and movement visualization, is the possibility of monitoring the frequency and quality of individual exercises and the possibility of a tele-rehabilitation intervention. The algorithms development for evaluating exercise quality is an up-to-date topic to solve. A minimally supervised rehabilitation program can lead to successful rehabilitation in motivated patients and those who do not have the opportunity for individual therapy under the supervision of a physiotherapist [26,27]. For this study, we also purposively selected a facility that would allow therapy to be conducted independently at home, be monitored remotely by health professionals, and meet the requirements for individualized treatment.

Therapy on a soft balance mat also had an effect in balance therapy. The advantage of this aid is its availability, reasonable price, and safety of use. Its disadvantage is the impossibility of visual inspection and lack of possibility of correction by an online specialist. 

The main limitations of this study include the different choices in balance aids used. It would be advisable for this type of study to be conducted on the same type of balance aids. Furthermore, it is a matter of debate whether 3 weeks of therapy is sufficient to improve static or dynamic stability.

## 5. Conclusions

Home balance therapy on various types of equipment is beneficial for musculoskeletal pathology. The experimental wobble board allowing visualization of movement seems to be a possible alternative to home balance training for post-traumatic and postoperative knee conditions when biofeedback is advantageous to increasing patients’ motivation to perform exercises. The evaluation of the physiotherapy process using the ICS Balance Platform shows that the experimental wobble board exercises have a positive effect, especially on the patients’ dynamic stability. Future research should focus on the possibility of supporting exercise on a wobble board in the home environment with functional real-time interactions with physiotherapists and automatic evaluation of exercise quality. 

## Figures and Tables

**Table 1:** Differences between the study and control groups before and after the intervention.

ICS Parameter	Control Group	Study Group	*p*
EO (mm/s)	0.74 ± 2.41	−0.15 ± 2.12	0.472
EC (mm/s)	1.80 ± 3.00	−0.09 ± 2.81	0.020
OLT (mm/s)	−1.00 ± 17.85	−1.06 ± 6.72	0.348
FC (mm/s)	−3.96 ± 11.65	−0.09 ± 5.55	0.138
Tar (hits)	4.40 ± 4.69	10.57 ± 6.47	<0.001
Purs (mm)	−1.68 ± 2.19	−2.57 ± 1.63	0.010
EO_P (−)	−8.40 ± 28.24	−3.33 ± 27.33	0.503
EC_P (−)	−11.20 ± 18.33	−1.00 ± 20.40	0.059
OLT_P (−)	0.80 ± 13.82	−0.33 ± 7.18	0.300
FC_P (−)	4.80 ± 20.64	0.00 ± 12.87	0.500
Tar_P (−)	11.20 ± 11.66	27.33 ± 16.60	<0.001
Purs_P (−)	16.80 ± 21.93	25.67 ± 16.60	0.010
P_Tot (−)	14.00 ± 58.67	48.33 ± 46.91	0.019

Values are given as mean ± standard deviation. The description of each parameter corresponds Section 2.6. The *p*-value represents the hypothesis that the control and study groups are statistically similar.

**Table 2:** Comparison of the communication strategy.

ICS Parameter	Educated	Controlled	Assisted	*p*
EC (mm/s)	0.2 ± 2.1	0.8 ± 2.9	−0.1 ± 2.4	0.745
Tar (hits)	13 ± 7	12 ± 6	9 ± 4	0.442
Purs (mm)	−2.6 ± 1.2	−2.9 ± 1.7	−2.6 ± 1.9	0.893

Values are given as mean ± standard deviation.

## Data Availability

Data are available upon request from the corresponding authors.
